# CD98hc (SLC3A2) drives integrin-dependent renal cancer cell behavior

**DOI:** 10.1186/1476-4598-12-169

**Published:** 2013-12-21

**Authors:** Marina Poettler, Matthias Unseld, Kira Braemswig, Andrea Haitel, Christoph C Zielinski, Gerald W Prager

**Affiliations:** 1 Department of Medicine I, Comprehensive Cancer Center, Medical University of Vienna, Vienna, Austria; 2 Department of Clinical Pathology, Medical University of Vienna, Vienna, Austria

**Keywords:** Integrin, Renal cell cancer, CD98

## Abstract

**Background:**

Overexpression of CD98hc (SLC3A2) occurs in a variety of cancers and is suspected to contribute to tumor growth. CD98, a heterodimeric transmembrane protein, physically associates with certain integrin β subunit cytoplasmic domains via its heavy chain, CD98hc. CD98hc regulates adhesion-induced intracellular signal transduction via integrins, thereby, affecting cell proliferation and clonal expansion. Disruption of CD98hc led to embryonic lethality in mice (E 3.5 and E 9.5) and CD98hc −/− embryonic stem cell transplantation failed to form teratomas, while CD98hc over-expression in somatic cells resulted in anchorage-independent growth. However, it is unclear whether interference with CD98hc expression tumor cell behavior.

**Methods:**

Renal cell cancer cell lines have been used to determine the effect of CD98hc expression on cancer cell behavior using cell adhesion, cell trans-migration and cell spreading assays. Flow cytometric analysis was performed to study the rate of apoptosis after detachment or serum starvation. shRNA-lentiviral constructs were used to stably knockdown or reconstitute full length or mutated CD98hc. The role of CD98 as a promotor of tumorigenesis was evaluated using an in *in vivo* tumor transplantation animal model. Immunohistochemical analysis was performed to analyze cell proliferation and CD98 expression in tumors.

**Results:**

This report shows that CD98hc silencing in clear cell renal cancer cells reverts certain characteristics of tumorigenesis, including cell spreading, migration, proliferation and survival *in vitro*, and tumor growth *in vivo*. Acquisition of tumorigenic characteristics in clear cell renal cancer cells occurred through the integrin binding domain of CD98hc. A CD98hc/integrin interaction was required for adhesion-induced sustained FAK phosphorylation and activation of the major downstream signaling pathways PI3k/Akt and MEK/ERK, while overexpression of a constitutive active form of FAK rescued the CD98hc deficiency.

**Conclusions:**

In this study we demonstrate that loss of CD98hc blocks tumorigenic potential in renal cell cancer.

## Background

Cytotoxic therapy is the only treatment available for many malignant diseases. However, molecular target therapies have recently become an additional and/or alternative therapeutic option. Biologic treatment is thought to be more specific, thereby resulting in fewer side effects. Renal cell cancer is a prominent representative for the efficiency of molecular target treatment, and led to the introduction of bevacizumab, sorafinib, sunitinib, pazopanib, and axitinib for the treatment of this disease [1-3].

Considering that not all patients respond to this kind of treatment, and those who benefit may become resistant [4,5], a better understanding of molecular mechanisms involved in specific tumor cell behavior is a requisite for efficient new therapeutic strategies. For example, the determination of biomarkers such as protein expression profiles might predict treatment response or prognosis. In this context, we described the heavy chain of CD98 (CD98hc), a type II transmembrane protein, as a biomarker for less differentiated and more aggressive renal cell cancers. We identified CD98hc as a marker of less differentiated clear cell renal cell cancer (ccRCC, G2-4) as well as of the less differentiated and more aggressive type 2 papillary renal cell cancer (pRCC). The benign renal tumors such as oncocytomas do not express CD98hc [6]. Under physiological conditions, CD98hc expression is limited to proliferating cells and activated T cells. CD98hc is mandatory for development, as genetic knock-out mice are embryonically lethal between day E 3.5 and E 9.5 [7,8]. Moreover, CD98hc −/− embryonic stem cell transplantation failed to form teratomas in mice [7]. I*n vitro* inhibition of CD98hc led to reduced cell growth and the induction of apoptosis in certain cell types, while overexpression of CD98hc in CHO cells resulted in anchorage-independent growth [9].

A functional role of CD98hc has been described in somatic cells where the cytoplasmic tail of beta integrin adhesion receptors was prerequisite for adhesion-induced signal transduction and integrin-mediated cell behavior in embryonic stem cells and fibroblasts [10-14]. In detail, CD98hc binds to a highly conserved C-terminal domain of integrin β1A and β3 cytoplasmic subunits, thereby affecting the integrin signaling cascade. In contrast, CD98hc does not interact with integrins β1D or β7 [12]. Furthermore, clustering CD98hc activates multiple integrin-dependent functions and mimics β1 integrin co-signaling in T-cells. Although cell adhesion is dispensable for both tumor cell- survival and -proliferation, mutation in beta integrins disrupts tumorigenesis [15]. Furthermore, deletion studies of integrins have demonstrated that the extracellular domain of integrins is dispensable, while the cytoplasmic domain is essential for tumor growth [15-17]. This is consistent with our previous findings that CD98hc directly interacts with the cytoplasmic domain of β1 or β3 tails [18]. The light chain of CD98 reconciles amino acid transport activity [19] and is covalently linked via disulfide bridges to CD98hc. The heavy chain is thereby essential to traffic the CD98 light chains to the cytoplasmic membrane [20].

Based on our recent data, we hypothesized that high expression of CD98hc influences malignant tumor cell behavior. We identified that CD98hc mediates tumor transplant growth *in vivo*. Utilizing both, gain and loss of function experiments *in vitro*, we also found that CD98hc is a major regulator of tumor cell behavior, thereby affecting tumor cell migration, proliferation, spreading and survival *in vitro.* The integrin-interacting domain of CD98hc was thereby crucial as truncation mutants were incapable to rescue CD98hc deficiency. Our data provides the first evidence that a biomarker, which is consistently over-expressed in high malignant renal cell cancers, bears a central functional role in integrin-dependent signal transduction and tumor cell behavior.

## Results

### CD98hc expression affects RCC growth *in vivo*

**Figure 1:**
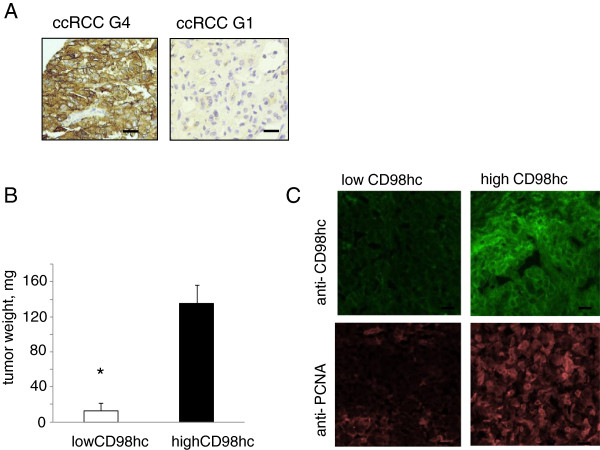
**CD98hc expression is correlated with grading of clear cell renal cell cancer (ccRCC) and affects tumor growth *in vivo*. (A)** Immunohistochemical staining for CD98hc in clear cell renal cell cancer. Left: high CD98hc expressing grade 4 ccRCC; right: no immunoreactivity of the anti-CD98hc mAB in grade 1 ccRCC. **(B)** In vivo tumor transplantation assays: Tumor weight (mg), lowCD98hc/Caki2 tumors, highCD98hc/Caki2 tumors. (* p< 0.0001, data represent mean ± S.D. of five mice per group after 8 days) **(C)** CD98hc expression was analyzed via immunofluorescence staining of low and highCD98hc/Caki2 cell tumors, grown for 8 days in the right flank of nude mice. Upper left: anti CD98hc in low CD98hc/Caki2 tumors, lower left: anti - PCNA staining in lowCD98hc/Caki2 tumors, upper right: anti C98hc in highCD98hc/Caki2 tumors, lower right: anti – PCNA in high CD98hc/Caki2 tumors. Size bars 200 μm.

Previously, we observed a direct correlation between CD98hc expression in clear cell renal cell cancer (ccRCC) and grade of differentiation [6]: We now analyzed CD98hc expression in paraffin-embedded tumor tissue sections derived from 51 ccRCC patients: The aggressive and less differentiated G3 and G4 ccRCC tumors were characterized by high CD98hc (G3: 87.0%; G4: 100.0%) expression, while the more differentiated G1 ccRCC revealed no CD98hc expression (G1: 0.0%). G2 had infrequent CD98hc expression (64.7%) (Figure 1A). [Qui square test: p = 0.007, n = 51]. Beside tumor cells, endothelial cells and lymphocytes revealed a positive immunostaining for anti-CD98hc. Although CD98hc has been suggested to affect cell behavior of somatic cells such as vascular smooth muscle cells [21], keratinocytes [22] or embryonic stem cells [7] most likely via integrins [18], any regulatory role of CD98hc is renal cell cancer cells has hitherto been unknown.

Thus, we generated a stable low CD98hc expressing ccRCC cell line (lowCD98hc/CaKi2) as well as a control high CD98hc expressing ccRCC cell line (highCD98hc/CaKi2) by the use of a CD98hc mRNA targeting shRNA and scrambled control bearing lentiviral construct, respectively. A PLKO-puro1 construct, lentiviral infection of CaKi2 cells led to stable downregulation of CD98hc to 5 ± 2% of highCD98hc/Caki2. When each stable cell lines were transplanted into athymic nude mice, a significantly enhanced tumor growth was only observed in the CD98hc over-expressing tumor transplants on day 8 (135 ± 21 mg in highCD98hc/Caki2 vs. 12 ± 9 mg in lowC98hc/Caki2 tumors, n = 5 mice/group) (Figure 1B). This was reflected by the enhanced immunoreactivity of an antibody against the proliferating cell nuclear antigen (PCNA), which represents a proliferation marker, in the highCD98hc expressing tumors cells, while the immunoreactivity in the lowCD98hc/CaKi2 transplants was faint (Figure 1C).

### Regulation of CD98hc expression in clear cell renal cancer cells

Having shown that expression of CD98hc expression is accompanied by tumor growth, we aimed to analyze causal underlying subcellular mechanisms. Various siRNA constructs were synthesized and applied in established renal cell cancer cell lines, such as CaKi1, CaKi2 or A-498 [20]. Although highest transfection efficacy was obtained in CaKi2 cells, key experiments revealed consistent results in all cancer cell lines used.

**Figure 2:**
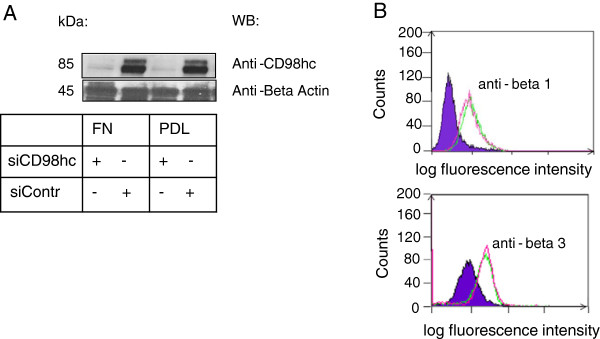
**CD98hc expression is not affected by integrins. (A)** Western blot (under reducing conditions). Matrix independent CD98hc expression, 85 kDa. Loading control = beta actin, 45 kDa. FN = cells seeded on fibronectin for or PDL (poly – D – lysine). Representative blots are demonstrated. 4 separate experiments. **(B)** FACS analysis demonstrate no alteration in beta 1 (upper pattern) or beta 3 (lower pattern) integrin adhesion receptors in low and high CD98hc expressing Caki2 cells.

CD98hc is a known interaction partner of integrin beta 1 and beta 3 subunits. However, regulation of CD98hc expression was not affected by integrin engagement, because in Caki2 cells seeded on the β1/β3 integrin ligand fibronectin as well as the artificial non-integrin ligand poly-D-lysine CD98hc was efficiently downregulated via siRNA (Figure 2A). Importantly, integrin β1/β3 expression were not affected by CD98hc regulation as were their ligand binding activity not altered. WOW-1 immunoreactivity, a monoclonal antibody specifically recognizing active integrin αvβ3, was not affected (lowCD98hc/Caki2 = 70 ± 3% of control, highCD98hc/Caki2 = 67 ± 8% of control, p = 0.12, n = 3).

### CD98hc regulates integrin-dependent cell functions in ccRCC

**Figure 3:**
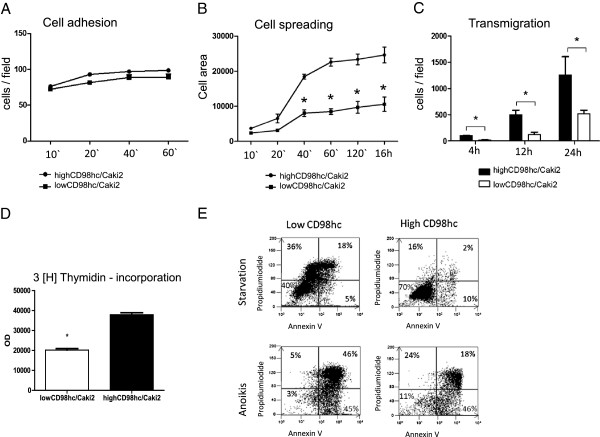
**Down-regulation of CD98hc results in reduced migration, proliferation and survival in Caki2 cells. (A)** Cell adhesion: Low and highCD98hc/Caki2 cells were seeded for 10, 20, 40 and 60 min on 10 μg/ml fibronectin and stained with crystal violet. Cells were counted per field with 20x magnification (n= 3, data represent the mean ± SEM, p=0.753), (1 field = 0.5 mm^2^). **(B)** Cell spreading: After attachments to 10 μg/ml fibronectin spreading was measured after 10, 20, 40 and 60 min. n=3, data represent the mean ± SEM, *p<0.001 **(C)** Cell transmigration: cell transmigration was analyzed using a modified Boyden chamber system, which revealed diminished migratory capability in lowCD98hc/Caki2 cells (white bars: mean 20 ± 3 cells/field after four hours, mean 122 ± 46 cells/field after 12 h and 520 ± 67 cells/field after 24 h) when compared to highCD98hc/Caki2 cells (black bars: mean 103 ± 5 cells/field after four hours, mean 495 ± 89 cells/field after 12 h and 1257 ± 346 cells/field 0.5 mm²) **(D)** Cell proliferation: cell [^3^ H] thymidine incorporation was reduced to 52 ± 3% after 24 h incubation under normal medium conditions in lowCD98hc/Caki2 cells when compared to control (highCD98hc/Caki2). n = 3, data represent the mean ± SEM, * p < 0.001. **(E)** Cell survival: Apoptosis induced by serum starvation or anoikis was measured via Annexin V/PI – FACS analysis, which revealed that lowCD98hc/Caki2 cells were more prone to apoptosis. Upper left panels point out necrotic cells. Representative dot blots given; n=5.

As CD98hc is whether affecting neither integrin expression (Figure 2B) nor its ligand binding capacities (see above), it was expected that regulation of CD98hc expression had no effect on Caki2 cell adhesion towards the integrin -β1/-β3 matrix protein fibronectin (Figure 3A). While it was previously reported that CD98hc does not affect integrin activation (inside-out) (Feral 2005), there is increasing evidence that CD98hc is a major mediator of adhesion-induced intracellular signal transduction via integrins (outside-in) [7,18]. Thus, we next analyzed potential effects of CD98hc in integrin-specific cell behavior in ccRCC cells.

LowCD98hc/Caki2 cells had an impaired cell spreading behavior when seeded on the integrin -β1/-β3 matrix fibronectin (Figure 3B), an effect which engages integrin-induced Rac and CDC42 activation [23]. Furthermore, ccRCC cell transmigration was affected by CD98hc expression, because the 4 hours transmigration rate of lowCD98hc/CaKi2 cells was decreased to 19 ± 2%, 24 ± 7% after 12 hours and 41 ± 4% after 24 hours when compared to highCD98hc/Caki2 cells (Figure 3C).

Tumor cell proliferation is another hallmark in tumor propagation. As the *in vivo* tumor proliferation analysis (Figure 1C) suggested a proliferation dependency on CD98hc expression, we were next interested in a potential regulation of CD98hc in ccRCC cell proliferation *in vitro*. By a 3H-thymidin incorporation assay a reduction to 52 ± 3% after 24 h was observed in lowCD98hc/Caki2 (Figure 3D). A prerequisite of metastasis formation is adhesion independent cell survival (anti- anoikis) as well as survival upon serum-starvation. High CD98hc/Caki2 cells were characterized by sustained cell survival, while low CD98hc expressing Caki2 cells were more prone to anoikis or apoptosis (Figure 3E).

From these data we conclude that CD98hc expression is essential for malignant ccRCC behavior including cell spreading, cell migration, cell proliferation and cell survival.

### The cytoplasmic domain of CD98hc is responsible for malignant tumor growth *in vivo*

**Figure 4:**
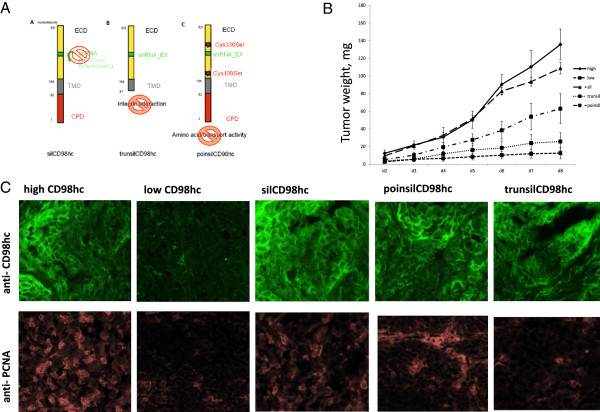
**The cytoplasmic domain of CD98hc is responsible for malignant tumor growth *in vivo.*** Reconstitution of CD98hc omitting shRNA binding was performed utilizing a QuickChange Kit (Stratagene) for the silent mutation (silCD98hc in **A**); a cytoplasmic truncation mutant was used to interfere with the integrin interaction (TrunSilCD98hc in **B**) and point mutations in Cys109 and Cys330 interfered with amino acid transporter interaction (poinsilCD98hc in **C**). Constructs were cloned in pcDNA 3.1 Vector via ECO RI. (ECD: extracellular domain, TMD: transmembrane domain, CPD: cytoplasmic domain). **(B)** Tumor weight (in mg), highCD98hc/Caki2 tumors, silCD98hcCaki2 tumors, lowCD98hc/Caki2 tumors, trunsilCD98hc/Caki2 tumors, poinsilCD98hc/Caki2 tumors. * p < 0.0001. Data represent means ± S.D. of three mice per group. **(C)** CD98hc expression was analyzed via immunofluorescence staining of lowCD98hc, highCD98hc, silCD98hc, trunsilCD98hc and poinsilCD98hc Caki2 cell tumor transplants, grown for 8 days after injection into the right flank of nude mice. Upper panel indicate anti CD98hc staining, lower panel indicate anti - PCNA staining.

To further elucidate whether interaction of CD98hc with the amino acid transporter (light chains of CD98), which engages the extracellular domain of CD98hc, or interaction with integrin adhesion receptors, which engages the cytoplasmic domain of CD98hc, we introduced CD98hc reconstitution mutants: (i) first, a silent mutation escaping shRNA binding capacities, thus, reconstituting for wild type CD98hc (silCD98hc) (ii) a silent mutation plus a truncation mutant lacking the cytoplasmic domain of CD98hc (trunsilCD98hc), which is expected not to interact with integrins [7] (iii) a silent mutation plus exchange mutations of the cysteins, thus, lacking CD98hc interaction with its light chains (poinsilCD98hc) (Figure 4A). Equally expression of silent mutations was confirmed by FACS analysis and again integrin beta 1 and beta 3 expressions were not affected by these mutations (data not shown).

By stable expressing these mutants in lowCD98hc/CaKi2 cells, we tested the functional role of CD98hc *in vivo* using tumor transplant assays. Reconstitution of wild type CD98hc in lowCD98hc/Caki2 by silCD98hc led to a similar rate in tumor growth as compared to highCD98hc/Caki2.

The single point mutations, lacking interaction with the amino acid transporters, only partly reconstituted for tumor growth, while the reconstitution with the truncation mutant lacking integrin interaction (trunsilCD98hc) failed to improve the tumor growth rate (Figure 4B).

Time-dependent tumor growth was consistently accompanied with immunoreactivity of an anti-PCNA antibody binding, reflecting cell proliferation *in vivo* (Figure 4C). From these data we conclude that CD98hc is essential for efficient *in vivo* tumor growth, whereby the cytoplasmic domain of CD98hc, which is thought to interact with integrin cytoplasmic domains thereby mediating adhesion induced signaling transduction is essential, while interaction with the CD98 amino acid transporter only partly contributed to efficient tumor growth.

### The cytoplasmic domain of CD98hc is essential for integrin-induced ccRCC cell behavior

**Figure 5:**
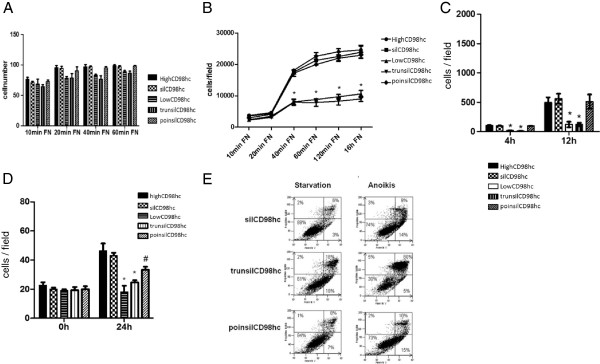
**Reconstitution of CD98hc rescues the rates of migration, proliferation and survival in low CD98hc CaKi2 cells. (A)** Cell adhesion in reconstitution mutant expressing CaKi2 cells: Adhesion was slightly, but not significant reduced in trunsilCD98hc Caki2 cells. silCD98hc expression or poinsilCD98hc expression showed no relevant correlation in cell adhesion. (n = 4, data represent the mean ± SEM, p > 0.05). **(B)** Spreading: Reconstitution with silCD98hc rescued the low CD98hc cell spreading phenotype starting after 20 min. However, the deletion mutant trunsilCD98hc Caki2 cells could not rescue reduced spreading at any time point, but had a similar spreading behavior as lowCD98hc/Caki2 cells. poinsilCD98hc/Caki2 cells had a similar rescued spreading effect as silCD98hc reconstitution in Caki2 cells. n = 3, data represent the mean ± SEM, * p < 0.001 **(C)** Transmigration of cells expressing reconstitution mutants: Transmigration in a modified Boyden chamber system revealed diminished migratory capability in trunsilCD98hc/Caki2 cells (mean 13 ± 2 cells/field after 4 h), compared to silCD98hc Caki2 cells (mean 100 ± 7 cells/field after 4 h) as well as to poinsilCD98hc Caki2 cells (mean 99 ± 3 cells/field after 4 h). n = 3, mean ± SEM, * p < 0.001 **(D)** Cell proliferation in reconstitution mutants: Reconstitution of silCD98hc was able to rescue the proliferative phenotype of low CD98hc expressing cells. trunsilCD98hc/Caki2 cells had similar proliferative activity such as lowCD98hc/Caki2 cells, whereas poinsilCD98hc/Caki2 cells could partially reconstitute for cell proliferation, however, the lower proliferative activity compared to highCD98hc/Caki2 was statistical not significant. n = 4, mean ± SEM, * p < 0.001, # > 0.05 **(E)** Survival in reconstitution mutant expressing cells: Apoptosis assay measured via Annexin V/PI– FACS analysis. Upper left panels reflect necrotic cells. Upper left panels reveal necrotic cells. Representative dot blots given; n=3.

Next, the respective mutations were stably expressed in the low CD98hc ccRCC cell line CaKi2 using a retrovirus (pBABE). Stable cell lines were analyzed for cell adhesion properties on the integrin -β1/-β3 ligand fibronectin. Neither high nor low CD98hc Caki2 nor the mutant expressing Caki2 cell lines revealed significantly altered adhesion cell behavior (Figure 5A).

When we tested, however, for integrin signaling-dependent cell spreading, we found that the low as well as the trunsilCD98hc Caki2 cells had a diminished spreading behavior. TrunsilCD98hc expression in Caki2 cells, which lack CD98hc interaction with the integrin β tails, could not rescue the integrin specific cell spreading behavior of lowCD98hc/Caki2. In contrast, poinsilCD98hc Caki2 cells had a similar spreading behavior as highCD98hc/Caki2 cells or silCD98hc/Caki2 cells (Figure 4B). Consistently, other integrin-induced cell behavior properties such as transmigration of CaKi2 cells were also dependent on the N – terminal domain of CD98hc, because trunsilCD98hc Caki2 cells could not reconstitute the high CD98hc phenotype (Figure 4C).

By studding either highCD98hc/Caki2 cells, lowCD98hc/Caki2 cells, silCD98hc/Caki2 cells, trunsilCD98hc/Caki2 or poinsilCD98hc/Caki2 cells for tumor cell proliferation, we found that only the wild-type CD98hc reconstituting mutant silCD98hc could completely rescue the high proliferative phenotype (93 ± 4% after 24 h and 98 ± 2% after 48 h of high CD98hc Caki2, p < 0.001). In contrast, the truncation mutant bearing cells (trunsilCD98hc/Caki2) had a similar proliferation character as lowCD98hc/Caki2 cells (trunsilCD98hc 49 ± 1% after 24 h and 53 ± 2% after 48 h of silCD98hc; lowCD98hc/Caki2 cells with 38 ± 3% after 24 h and 49 ± 5% of highCD98hc Caki2 cells after 48 h, p < 0.001 (24 h) and p < 0.001 (48 h)). The poinsilCD98hc/Caki2 cells, however, had a non-significant (p = 0.75) trend for a diminished proliferative activity when compared to highCD98hc/CaKi cells (77 ± 2% of silCD98hc after 24 h and 86 ± 4% of silCD98hc after 48 h). The cytoplasmic domain of CD98hc might thus play a major role in renal cancer cell proliferation most likely via previously described interaction with integrin β tails [12] (Figure 5D).

Finally, we tested the mutants for cell survival upon starvation as well as upon cell detachment. Thus, we found that silCD98hc as well as poinsilCD98hc could reconstitute for high CD98hc expression, while the truncation mutant trunsilCD98hc showed a similar apoptosis behavior as the low CD98hc Caki2 cells (Figure 5E). From these data we conclude that the cytoplasmic domain of CD98hc is essential for mediating tumor cell function as it affects cell spreading, cell transmigration, cell proliferation and cell survival.

### Cytoplasmic domain of CD98hc is responsible for integrin-induced signal transduction and cell spreading

**Figure 6:**
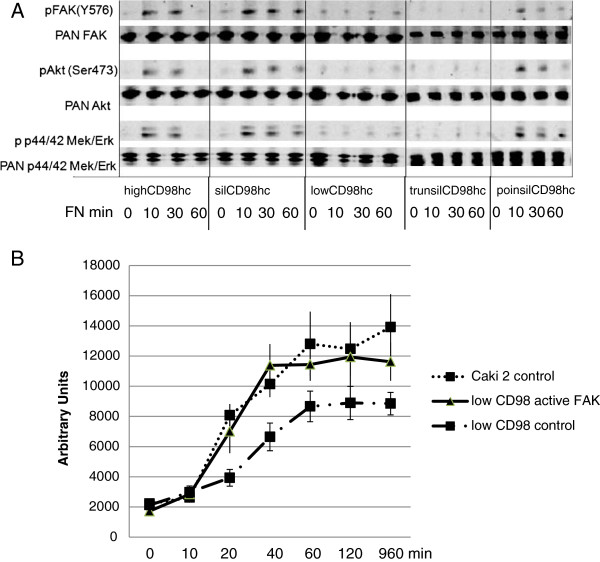
**The cytoplasmic domain of CD98hc regulates integrin-induced signal transduction and spreading. (A)** Signaling: CD98hc is a major contributor to integrin-dependent signal transduction involving pFAK, Akt as well as the MEK/ERK pathways. High and low CD98hc Caki2 cells as well as the mutated Caki2 cells silCD98hc/Caki2, trunsilCD98hc/Caki2 and poinsilCD98hc/Caki2 were permitted to adhere to fibronectin for time-spans indicated. Phosphorylation of FAK (lane 1) was compared to total FAK (lane 2) as well as phosphorylation of pSer Akt (lane 3) was compared to total Akt (lane 4). Furthermore, phosphorylation of p44/42 MEK/ERK (lane 5) was compared to total p44/42 (lane 6). Only the trunsilCD98hc Caki2 cells were unable to rescue the lowCD98hc/Caki2 effect on intracellular signal transduction. n = 3, data represent the mean ± SEM, * p < 0.001 **(B)** Cell spreading assays: highCD98hc/Caki2 and lowCD98hc/Caki2 cells were either transfected with dominant active FAK of the empty control vector. Cells were seeded on fibronectin (FN, 10 μg/ml) for indicated time spans before cell area was assessed. Bars indicate SEM- standard error of mean.

Adhesion-dependent FAK activation leads to induction of downstream signaling pathways such as MEK/ERK, a major driver pathway for cell proliferation, or PI3K/Akt, a major cell survival and motility inducer. Thus, we next analyzed whether the previously suggested cytoplasmic domain of CD98hc is also responsible for adhesion-induced signal transduction in the cancer cell line CaKi2. We found, that silCD98hc as well as poinsilCD98hc could rescue adhesion-induced Y576 FAK phosphorylation, while the low CD98hc as well as trunsilCD98hc mutants did not lead to an increase in phosphorylation of FAK Y567. Consistently, major downstream signaling pathways such as PI3kinase/Akt as well as MEK/ERK were only activated upon cell adhesion on fibronectin whenever the cytoplasmic domain of CD98hc was present. This effect was independent of the cysteine sides described for light chain interaction (Figure 6A).

To analyze whether integrin-induced signal transduction is indeed responsible for CD98hc-dpendent tumor cell behavior, we next overexpressed dominant active FAK. Integrin-induced FAK phosphorylation was shown to be dependent on the direct interaction of CD98hc with a conserved domain of the cytoplasmic tail of beta-1 integrins [18], thus overexpression of active FAK is thought of rescue CD98hc deficiency. highCD98hc/Caki2 or low highCD98hc/Caki2 were transiently transfected with dominant active FAK or empty vector. While with the control vector transfected lowCD98hc/Caki2 cells had a significant reduced spreading phenotype when compared to the control vector infected highCD98hc/CaKi2 cells, active FAK was capable to rescue for CD98hc deficiency. These data suggest FAK as a downstream effector of CD98hc in tumor cell behavior (Figure 6B).

## Discussion

Recent advances in our understanding of the complex molecular mechanism for malignant transformation, tumor growth, and propagation have led to a much more complex set of challenges for diagnostic or therapeutic strategies than originally anticipated. Among the drivers of malignant growth, receptor tyrosine kinases, functional activating molecular mutations, as well as integrin adhesion receptors are thought to be the major contributors of tumorigenesis. In this context the integrin-interacting molecule CD98hc, a type II transmembrane glycoprotein, was demonstrated to lead to malignant transformation, whereby CD98hc acted as an oncogene stimulating molecule leading to anchorage– independent growth within CHO cells [9].

Further evidence of the pivotal role of CD98hc in malignant growth was provided by an embryonic stem (ES) cell transplantation model, whereby only wild type CD98hc expressing embryonic stem cells formed teratomas, while CD98hc deficient embryonic stem cells showed a significant reduction in proliferation as well as reduced cell survival characteristics, thereby leading to diminished tumor growth [7]. We recently demonstrated that CD98hc expression is mainly found in the less differentiated and more aggressive renal cell cancer subtypes such as type II papillary renal cell cancer or clear cell renal cell cancer [6]. Our analysis of tumor tissue sections from ccRCC patients demonstrated a significant correlation between CD98hc expression and grade of malignancy (Figure 1A).

Based on these and previous observations, we hypothesized that CD98hc is a major driver of malignant tumor cell behavior and aimed to characterize any pivotal functional role of CD98hc in tumor cell biology. Thus, we first generated either high expressing CD98hc or very low CD98hc expressing ccRCC cell lines to analyze malignant cell behavior as well as the molecular pathways responsible for CD98hc mediated tumor cell functions.

In order to test whether CD98hc plays a role within renal cell carcinoma biology, we xeno-transplanted highCD98hc/Caki2 cells or lowCD98hc/Caki2 cells into nude mice. We observed a striking difference in tumor growth and subsequent loss of anti-PCNA immunoreactivity. This suggested that CD98hc regulates tumor cell proliferation (Figure 1B). Moreover, we were able to exclude any effect of CD98hc on integrin expression or integrin-ligand binding capacities (Figure 2B, Figure 3A). This is consistent with previous observations that CD98hc does not affect integrin expression or activation [7], but mediates adhesion-induced signal transduction (outside-in signaling). This is most likely induced by direct interaction of the cytoplasmic domain of CD98hc with a conserved motif of the C-terminal end of integrin beta-tails, which is a prerequisite for efficient FAK phosphorylation upon cell adhesion [18]. Although not proven, it is tempting to speculate that CD98hc might promote complex formation of most upstream signaling molecules such as member of the src-family kinases, FAK or others. We were interested whether CD98hc-dependent tumor growth *in vivo* was mediated via integrin-induced signal transduction (outside-in) or CD98hc-dependent amino acid transportation via its light chains. When we first examined integrin-mediated cell behavior, such as cell spreading, cell migration or anoikis, the so called apoptosis upon cell detachment, we found that all these cell functions were dependent on CD98hc-expression. This was an important observation as it was unclear whether these tumor cell functions were affected by CD98hc. Our data suggest that CD98hc, when over-expressed, augments malignant cell behavior, such as tumor cell spreading, transmigration, proliferation, or cell survival. All these functions are thought to be hallmarks for circulating tumor cell survival and metastasis formation.

Although integrin-dependent cell functions were strongly affected by CD98hc expression, were still interested whether the cytoplasmic integrin-interaction domain [9,18] or the cysteine-bridges to the amino-acid transporting light chains of CD98hc were dispensable for malignant tumor cell behavior. We generated silent mutations, which were not recognized by shRNA constructs, in order to rescue CD98hc expression in lowCD98hc ccRCC cells. We also introduced either cytoplasmic domain-truncation mutants (trunsilCD98hc) or mutants which lack the light chain interaction (poinsilCD98hc). We found that the major steps of malignant cell behavior were dependent on the cytoplasmic tail of CD98hc, among them tumor cell migration, cell spreading, cell proliferation as well as cell survival. In contrast, the amino acid transport activity partly affected cancer cell proliferation *in vitro* and *in vivo*, which was not statistically significant (p-value high CD98hc vs. poinsilCD98hc p = 0.12). Finally, our *in vitro* findings were also reflected by *in vivo* tumor transplantation assays (Figure 4).

One could speculate that the interaction partners for CD98hc beta 1 integrins as well as beta 3 integrins, which are both expressed in CaKi2 cells and have previously been shown to interact with CD98hc [9,10], were mediating adhesion-induced signal transduction via induction of FAK and c-src whenever CD98hc was present. This is also supported by the fact that whenever the cytoplasmic domain of CD98hc for integrin binding was absent, for instance in CD98hc reconstituted cells, a diminished FAK phosphorylation upon cell adhesion on a beta 1 integrin as well as beta 3 integrin-specific matrix protein was observed.

Furthermore, overexpression of dominant active FAK rescued the low CD98hc spreading phenotype. This is of special importance in respect to previous findings that malignancy of certain tumor cells depends on activation of upstream integrin signaling events [11]. This is also consistent with our *in vivo* findings that the absence of the cytoplasmic domain of CD98hc, which was demonstrated to be responsible for efficient integrin-induced signal transduction [10], led to a significant reduction in tumor growth. In a previous study, we analyzed CD98hc expression in various tumor cell lines and found that CD98hc is frequently expressed in aggressive tumor cells derived from adenocarcinomas of the lung, colon and breast [6]. Therefore, CD98hc expression is significantly associated with more aggressive and less differentiated G3, G4 ccRCC (Figure 1A) and supports the observation of an enhanced activation state within tumor cells. Whether ccRCC metastasis formation generally depends on CD98hc expression was not the focus of this study and is currently being investigated.

## Conclusion

In conclusion, by a combination of different *in vitro* and *in vivo* attempts, we aimed to define a potential functional role of CD98hc in renal cell cancer. We observed a correlation between less differentiated and more aggressive clear cell renal cell cancer and CD98hc expression. We found that CD98hc is not only a descriptive marker for aggressive cancers, but bears a major regulatory role of malignant cell function. This was demonstrated by knock down and reconstitution *in vivo* and *in vitro*, thereby suggesting that the integrin interacting domain of CD98hc is required for the malignant phenotype of renal cancer cells. It is tempting to speculate, that these novel insights will lead to more effective strategies in cancer treatment.

## Methods

### Cell culture

Caki-2 cells were cultured in RPMI 1640 (10% FBS and 5% Penicillin/Streptomycin). 24 hours prior to experiments cells were maintained in antibiotic free medium under serum reduced conditions (5% FBS). Experiments were performed under serum-free conditions.

### Downregulation of CD98hc and production of lentiviral particles

CD98hc siRNA was purchased from Santa Cruz-Biotechnology and used according to manufactures instructions. Primers for shRNA CD98hc were purchased from Invitrogen. Annealed oligos were cloned AgeI/EcoRI to pLKO-puro1. pLKO.1 TRC Cloning Vector and reagents were used from Addgene and carried out according to the manufactures guidelines. Cells were coated on polyprene (10 μg/ml) prior to lentiviral application, medium was changed after 24 hours and protein quantification was performed after 48 hour. For generation of stable lowCD98hc or highCD98hc/Caki2 (scrambled shRNA) cells were grown in the presence of puromycin (5 μg/ml) for at least two weeks.

### Mutations

Reconstitution of CD98hc by silent mutations was performed utilizing a QuickChange-Kit (Stratagene) using a pcDNA 3.1 Vector. The cytoplasmic truncation mutant (trunsilCD98hc), the deltaWALLL truncation nucleotide 1–87, has been described before [9] and was generated to interfere with integrin interaction. The Cys109 and Cys330 (poinsilCD98hc) were performed as described by Fenczik, 2001 [20]. Constructs were cloned in pBABE via ECO-RI [20,24]. All numbering uses the amino acid sequence reported in entry 4F2_human (P08195-1) of the Swiss-Prot data base as of January, 2010.

Transient transfection of HEK-293 cells and retroviral infection of lowCD98hc Caki2 cells were performed as described before [25]. Stable infectants were selected in growth medium containing 1 mg/ml puromycin.

### Proliferation assays

#### MTT-test

50 μl normal medium was transferred in 96-well plate and incubated at 37°C. Cells were harvested with Trypsin/EDTA and cell number was counted. 5000cells/well were transferred in 96-wells and incubated for 24 or 48 hours. 15 μl Dye solution was then added and again incubated 4 hours. 100 μl Solubilisation-/Stop-solution was added and after 2 hours at room temperature absorption was measured in a photometer at 750 nm.

#### Counting – assay

Cells were harvested with trypsin/EDTA as described above; 1000, 2000 or 5000 cells were transferred in each plate (96well) before cells of 10 fields of a raster ocular (1 field = 1 mm^2^) were counted under light microscope after one hour adhesion time and counted again after 24 and 48 hours.

#### Thymidine incorporation

Different concentrations of lowCD98hc/Caki2 cells or control cells were seeded in a 96well plate with medium (RPMI + 10% FCS + 1% P/S). After 18 h cells were treated with 50 μl per well thymidine (H3 : RPMI = 1:10) 1 h, 37°C, 5%CO_2_ before cells were washed and lysed. Incorporated 3H-thymidine was then detected by liquid scintillation.

#### Cell transmigration

Cell migration was assayed in a modified Boyden chamber-system by using transwell membranes (8 μm) coated with 1% gelatin. Cells were seeded on the top of the membrane in medium without FCS, while in the lower chamber 10% FCS was added as a stimulus. After four hours filters were washes with PBS ×1, fixed (Methanol: acetic acid, 3 : 1) and nuclear stained with DAPI. Migrated cells in the lower chamber were counted using an AX70 Olympus-microscope and compared to controls (absolute numbers per mm^2^ is given).

### Cell survival

#### Anoikis- and starvation-assays

Poly 2-hydroxyethyl-methacrylate (polyHEMA) was used for detachment cell survival assays. PolyHEMA was diluted in 75% ethanol as described by manufacture, mixed and kept at 37°C overnight. One hour before experimental onset 110 μl of PolyHEMA was added in 24- well plates and air dried at room temperature. 1×10^6^ cells were then transferred in prepared 24- well plates and after 24 or 48 hours incubation time apoptosis upon detachment was measured. For cell starvation, 1% BSA for 24 or 48 hours at 37°C, 5%CO_2_ was used.

Cells were stained with Annexin V-FITC Detection Kit (Alexis Biochemicals, Farmingdale, NY) and propidium iodide to estimate apoptosis and necrosis via flow cytometry (FACSCalibur, BD).

#### Cell adhesion and spreading

Assays of cell spreading were either performed on 20 μg/ml fibrinogen (Fg) or 10 μg/ml fibronectin (FN) or 10 μg/ml Poly-D-Lysine (Millipore) as described previously [26]. The cells were allowed to attach for 30 minutes for adhesion assay before plates were washed with phosphate-buffered saline (PBSx1); attached cells were fixed with 3.7% formaldehyde and stained with crystal violet. Photographic images were acquired with Olympus SC20-CCD on a bright-field microscopy. Cells that exhibited flattening and the presence of lamellipodia under microscope examination were scored as spreading cells. Cell area was assessed by Image J1.32 software (National Institutes of Health).

#### Leucine uptake

[14C]- Leucine (50 μCi) was purchased from Perkin Elmer, normal L-leucine from Sigma. 2× 10^5^ lowCD98hc/- or highC98hc/Caki2 cells were washed tree times in a Na- free Solution (125 mM Cholin Cl, 4,8 mM KCl, 1,3 mM CaCl2, 1,2 mM MgSO4, 25 mM Hepes-Tris, 1,2 mM KH2PO4, 5,6 mM Glucose, pH 7,4) than 20 mM L-Leucine/[14C]- Leucine was added to Na- free solution for 1 min or 10 min, washed with Na free Uptake solution and lysed with 50 μl RIPA-Buffer. After transferring lysates in scintillation plates, 100 μl scintillator-liquid was added and [14C] - Leucine uptake was measured via a scintillator.

#### In vivo transplant assay

HighCD98hc/Caki2, lowCD98hc/Caki2, silC98hc/Caki2, poinsilCD98hc/Caki2 and trunsilCD98hc/Caki2 cells were xenotransplanted into 8 weeks old nude mice by subcutaneous injection (s.c.) into the right flank, five animals per group. After 8 days mice were sacrificed and tumors were extracted and embedded in mounting medium. 4 micrometer sections were stained immunohistochemically for 4'-6-diamidino-2-phenylindole (DAPI), CD98hc (C-20): sc- 7095 (Santa Cruz Biotechnology, CA) or PCNA (Abcam, UK).

## Patients

The study included 51 paraffin-embedded ccRCCs, from kidney origin. All tumors were derived from a well characterized tissue bank of the Department of Clinical Pathology, Medical University of Vienna. Specimens were sliced before they were routinely fixed overnight with 4.5% buffered formaldehyde and stained for CD98hc as described previously [6]. ccRCCs were classified according to the TNM-System (T = Tumor, N = Node, M = Metastases according to the Unio Internationalis Contra Cancrum, UICC) [27], and graded according to Fuhrman et al. [28]. None of the patients had been treated with chemotherapy, tyrosine kinase inhibitors or immunotherapy prior to surgery.

Detailed information about Material and Methods is located within Additional file 1.

## Competing interests

The authors declare no competing financial or conflicting interests.

## Authors' contributions

GWP, and MP developed the hypotheses. GWP, CCZ and MP wrote the manuscript. GWP designed the experiments and evaluated the data. MP, MU and KB executed most of the experiments. AH provided the transgenic tumor tissue samples and critically red the manuscript. AH and MP provided pathological review of histology and immunohistochemistry. All authors read and approved the final manuscript.

## Supplementary Material

Additional file 1 Supplemental Materials and Methods. Click here for file
