# Supplementary material for: CD98hc (SLC3A2) drives integrin-dependent renal cancer cell behavior
Source: Mol Cancer. 2013 Dec 21;12:169. doi: 10.1186/1476-4598-12-169 (PMC3879186; doi:10.1186/1476-4598-12-169)
Supplement: Additional file 1 — Supplemental Materials and Methods. [file 1476-4598-12-169-S1.docx]

## Additional File 1

**Supplemental Materials and Methods**

Cell culture

Caki2 (ATCC® HTB47™) were maintained in a humidified atmosphere of 5% CO2, 95% air, 37°C and grown in RPMI 1640 with L- Glutamine (PAA Laboratories, Pasching, A) supplemented with 10% Fetal Bovine Serum (Mycoplex) (PAA) and 5% Penicillin/Streptomycin (PAA). For sub- culturing cells were dissociated with 0,1% Trypsin/ EDTA (100X) (PAA), split and sub - cultured 1:5 in Costar flasks 75cm2 growth areas (Costar, Badhorvedorp, NE) and centrifuged with 300g for five minutes. The cell medium was changed every two days and cells reached confluence after 3 – 4 days of incubation. 24 hours prior to experiments cells were maintained in antibiotic free medium under serum reduced conditions (5% FBS). Experiments were generally performed under subconfluent conditions.

Downregulation of CD98hc and production of lentiviral particles

CD98hc siRNA was purchased from Santa Cruz Biotechnology (Target sequence of Hs_SLC3A2_1 siRNA: AATCCTGAGCCTACTCGAATC, Hs_SLC3A2_2 siRNA: GTTCAAGAGACTTCTCGGGCT, Hs_SLC3A2_3 siRNA: GTTCAAGAGACAAGAATGAGG)

and used according to manufactures instructions. Shortly, 2 x 10 5 cells/ 600 µl Medium were

plated in 24well plates, 75nMsiRNA were mixed with Serum free RPMI and kept for 30 min on room temperature. Then siRNA complexes were drop wise pipette to cells, after 6 hours 400µl RPMI containing 10% Serum and 1% Penicillin/Streptomycin was added. 48h later CD98hc expression was measured via FACS analysis.

Primer for shRNA CD98hc were purchased from Invitrogen. (Primer Forward: 5´- CCGGTAATCCTGAGCCTACTCGAATCttcaagagaGATTCGAGTAGGCTCAGGATTTTTTTG

–3´, Primer Revers: 3´-

ATTAGGACTCGGATGAGCTTAGaagttctctCTAAGCTCATCCGAGTCCTAAAAAAACTTAA-

5`) Annealed oligos are cloned AgeI/EcoRI to pLKO-puro1. 7 x 105 HEK- 293 cells in a 6cm culture flask with 5ml DMEM supplemented with 10%FCS were incubated on 37°C, 5% CO2, 95% air overnight. pLKO.1 TRC Cloning Vector Protocol and reagents were used from Addgene and carried out according to the manufactures guidelines. Cells were coated with polyprene (10µg/ml) prior to lentiviral application, medium was changed after 24 hours, and western blot analysis as well as FACS analysis were performed after 48 hour (described below). For the production of stable low/CD98hc or high/ CD98hc Caki2 (scrumbled shRNA) medium supplemented with Puromycin (5µg/ml )was changed every two days for at least two weeks.

Mutations

Reconstitution of the shRNA was performed utilizing a QuickChange Kit (Statagene) for this silent mutation. Forward primer (3`- CAG ATT TTA TCG TTG TTG GAG AGC AAC AAA GAC TTG CTG TTG A – 5`) and reverse primer (5`- TCA ACA GCA AGT CTT TGT TGC TCT CCA ACA ACG ATA AAA TCT G – 3`) were purchased from Invitrogen.

Cytoplascmic truncation mutants were used to interfere with the integrin interaction; deltaWALLL truncation nucleotide 1-87 is described by Songmin et al., 2004 and Zent et al., 2003. The silent mutation – reconstituted CD98hc construct (silCD98hc) will here serve as the template for generating mutations in pcDNA 3.1 Vector: trunsilCD98hc (forPrimer: 5’ AATCAGGATCCATGCTCTTCTGGCTCGGCTGGC 3’, Rev_primer_HA: 5’

AATCAGAATTCTCAGGCATAGTCTGGGACG 3’)

To interfere with amino acid transporter point mutations on Cys109 and Cys330 (poinsilCD98hc) were performed using QuickChange Kit (Statagene) described by Fenczik, 2001 [27]. Constructs are cloned in pBABE via ECO RI. [27, 32]

All numbering uses the amino acid sequence reported in entry 4F2_human (P08195-1) of the Swiss-Prot data base as of January, 2010.

CD98 heavy chain constructs were subcloned into pBABE retroviral vector (Clontech). Transient transfection HEK-293 cells and retroviral infection of lowCD98hc Caki2 cells was

performed as described [33]. Stable infectants were selected in growth medium containing 1 mg/ml puromycin.

Transformation

The competent DH5α cells (Invitrogen, Lofer, A), were thawn on ice and gently mixed with the pipette tip and aliquoted to 50μl of cells for each transformation into 1.5ml tubes that have been pre-chilled on ice. 1-5μl DNA (100ng) are added to the cells and mixed gently. Tubes were placed on ice for 30 minutes and heat shocked at 42°C for exactly 30 seconds without shaking. The tubes were then placed on ice for 2 minutes. After adding 250μl of pre-

warmed (37°C) SOC medium (Sigma- Aldirch, Saint Louis, MS, USA), tubes were shacked at 37°C for 1 hour. Tubes were centrifuged, supernatant was discarded and the pellet was resuspended in the small amount of the left supernatant and each transformation is spread onto AgarAgar (Sigma) plates with appropriate antibiotic (Ampicillin 1:1000 (Sigma)). Plates were allowed to dry and incubated inverted at 37°C overnight. Cones were then picked and resuspended in Luria Broth (Sigma) with the appropriate antibiotic (Ampicillin 1:1000 (Sigma)). Bacteria were allowed to proliferate for 8 hours at 37°C, shacking. Thereafter DNA was obtained using Quiagen mini or maxi prep – kits according to manufactures instruction. DNA concentration was measures using a NanoDrop (PeqLap).

Transfection

Caki2 cells were transfected with pcDNA 3.1 (Invitrogen) containing the various CD98hc constructs using Lipofectamine LTX according to manufactors instructions (Invitrogen). 5x105 cells were plated the day before transfection in 6 well plates with RPMI supplemented with 10% FCS without antibiotics and incubated at 37°C, 5%CO2, 95% air. For positive selection 500 µg/ml geneticine (PAA) was used.

Western Blot

Cells were washed with D- PBS (Dulbeccos) (PAA), lysed with RIPA Buffer and scrabed with a cell scraper, kept 30 minutes on ice with vortexing every five minutes and centrifuged 10 minutes at 14000rpm and 4°C. Supernatant was taken and Lammeli Buffer (Sigma) was added in a 1 : 1 ration. Lysates were than cooked at 95 °C for 10 minutes. Lysates were applied on 4% Tris – HCl Gels (BioRad), For blotting power supply war held on 120 V for 90 minutes and 4°C. Membrane was than kept in Blocking Buffer (100ml Tris buffered Saline (TBS)(0,5M Tsis – HCl, 0,9% NaCl, pH=7,4), 3%BSA, 0,1% Tween 20)over night. First antibody was diluted in desired concentration in TBS with 1% Tween 20and 3% BSA and incubated for two hours on a shaker. Antibodies uses : Anti CD98 (C-20): sc- 7095 (Santa Cruc Biotechnology, Inc. , Santa Cruz, CA, USA), anti-phospho- Akt (Ser473) ( 1:1000), anti beta- Actin Antibody , Anti p44/42 MAP Kinase ( 1:1000) , anti phosphor p44/42 MAP Kinase (Thr202/thr294) ( 1:1000), anti phosphor 4E-BP1 (Thr37/46) ( 1:1000), anti phosphor p70 S6 Kinase (Thr421/Ser424) ( 1:1000), anti FAK [pY576] (Santa Cruc Biotechnology, Inc., Santa Cruz, CA, USA) ( 1:1000). Membrane was than washed 30 minutes by changing TBS with 1% Tween 20 all five minutes. Secondary antibodes IRD dye (Rockland) either for rabbit, goat or mouse (700 or 800nm) were used in a concentration of 1:10000 and incubated for one hour, shacking, afterword the washing procedure was repeated. Antibody detection was then performed on an Odysee, Licor.

FACS Analysis

Cell were harvested in Trypsin / EDTA centrifuged, resuspended in PBS 3% BSA and washed tree times in PBS 3% BSA, first Antibodies (Anti CD98hc (C-13), 1:500), Anti PCNA

- Proliferation Marker (Abcam, Cambridge, UK) (1:300), Anti WOW Fab (a kindly Gift from S. Shattil) (1:00) or FITC CD98hc (1:100), Anti β1 Integrin (Huts-4) ( 1:300),Anti Human Integrin β3 (GPIIa, CD61) ( 1:500), Anti αVβ3 (Vitronectin receptor) (LM 609) ( 1:100) (Chemicon International, Millipore), labeled antibody were incubated 30 minutes on ice. Cells were than washed three times again with PBS 3% BSA, followed by 15 minutes second antibody (Alexa

Fluor 488 F(ab`)2 fragment of goat anti- mouse IgG (H + L), Alexa Fluor 488 F(ab`)2 fragment of goat anti- rabbit IgG (H + L) (Invitrogen, Lofer, Sbg, Austria)) incubation, if necessary, and/or 5 minutes propidium iodide (Sigma) stain for apoptotic cell detection. Cells were washed again with PBS 3% BSA und resuspended in 500 µl PBS 3% BSA. A FACSCalibur (BD) was uses for flow cytometrical analysis.

Proliferation assays

MTT-test

50µl normal medium was tranferred in 96-well plate and incubated at 37°C. Cells were harvested with Trypsin/EDTA as described before and cell number was counted with Casy cell counter. 1x 105 cells were resuspended in 1ml medium, 50µl (5000 cells/well) were transferred in prepared 96-wells and incubated for 24 or 48 hours at 37°C, 5%CO2, 95% air. 15µl Dye solution was then added and again incubated 4 hours at 37°C, 5%CO2, 95% air. 100µl Solubilisation / Stop solution was added and after 2 hours wrapped in aluminum foil at room temperature absorption was measured in a photometer at 750nm.

Counting – test

Cells were harvested with trypsin/EDTA as described before, 1000, 2000 and 5000 cells were transferred in 96well plates with normal medium. Cells of 10 fields of a raster ocular (1 field = 1mm2) were counted under light microscope after one hour adhesion time and counted again after 24 and 48 hours.

Thymidin incorporation

Different concentrations of low/CD98hc/Caki2 cells as well as control cells were seeded in a 96well plate under normal conditions (RPMI + 10% FCS + 1% P/S). 18h after incubation cells were treated with 50µl per well Thymidin (H3 : RPMI = 1: 10) 1h, 37°C, 5%CO2, cells were

then washed three times with ice cold PBS and 100µl lysis-buffer was added. Lysates were filled in appropriate scintillation vials, wells were washed again with 100µl PBS, which was added to tubes. Incorporated 3H-thymidine was then detected by liquid scintillation.

Cell transmigration

Cell migration was assayed in a modified Boyden chamber system by using transwell membranes (8 µm) coated with 1% gelatin in 24well plates. Cells were seeded to the top of the membranein medium without FCS, while in the lower chamber 10% FCS was added as a stimuli . After four hours filters were washes with PBS 1x, fixed (Methanol: acetic acis, 3 : 1) and stained/ covered with 4'-6-diamidino-2-phenylindole (DAPI) for nuclear staining. Migrated cells in the lower chamber were counted using an AX70 Olympus microscope and compared to control (results are given per absolute number per unit (1 mm2).

Cell survival

Anoikis

Poly (2-hydroxyethyl methacrylate), polyHEMA, was used for detachment survival assays. PolyHEMA was diluted in 75% Ethanol as described by manufactor, mixed and kept at 37°C over night. One hour before experimental onset 110µl of PolyHEMA was added in 24- well plates and air dried at room temperature. 1x106 cells were then transferred in prepared 24- well plates and after 24 or 48 hours incubation time at 37°C, 5%CO2, 95% air.

Starvation

Cells were harvested as described before and washed 3 times with RMPI without FCS or antibiotics before 1x106 cells were transferred into 24 well plates with 300µl RPMI supplemented with 1% BSA for 24 or 48 hours at 37°C, 5%CO2.

Cells were then stained with Annexin V-FITC Detection Kit (Alexis Biochemicals, Farmingdale, NY,USA) and propidium iodide to estimate apoptosis, necrosis and viable cells via flow cytometry (FACSCalibur, BD).

Cell adhesion and spreading

Assays of cell spreading were either performed on 20µg/ml fibrinogen (Fg) or 10µg/ml fibronectin (FN) or 10µg/ml Poly-D-Lysine (Millipore) were performed as described previously

{Liu, 1999 617 /id}. The cells were allowed to attach for 30 minutes for adhesion assay. The spreading assay was performed in the same way, but cells were fixed after certain time points. Unattached cells were washed away with phosphate-buffered saline; attached cells were fixed with 3.7% formaldehyde and stained with crystal violet. Photographic images were acquired with Olympus SC20 CCD on a bright field microscopy. Cells that exhibited flattening and the presence of lamellipodia under microscope examination were scored as spreading cells. Cell area was assessed by Image J 1.32 software (National Institutes of Health).

Leucine uptake

[14C]- Leucine (50µCi) was purchased from Perkin Elmer, normal L-leucine from Sigma. 2x 105 lowCD98hc/- or highC98hc/ Caki2 cells were washed tree times in a Na- free Solution (125mM Cholin Cl, 4,8mM KCl, 1,3mM CaCl2, 1,2mM MgSO4, 25mM Hepes-Tris, 1,2mM KH2PO4, 5,6mM Glucose, pH 7,4) than 20mM L-Leucine/ [14C]- Leucine was added to Na- free solution for 1min or 10min, washed with Na free Uptake solution and lysed with 50µl Ripa-Buffer. After transferring lysates in scintillation plates, 100µl scintillator-liquid was added and [14C] - Leucine uptake was measured via a scintillator.

Cells or tumor tissue were fixed in formaldehyde for 24 hours. Before embedding the specimens in paraffin, they had to run through an ascending alcohol series (50%, 70%, 80%, and 96%). Specimens were cut with a microtom in four micrometer slices and these were then put on glass slides. Before staining tissue slides ware incubated in Xylol two times for 10 minutes, then they pass through a descending alcohol series (96%, 70%,50%) and H2O dest. , followed by a incubation in Methanol/H2O2 (3%) for 10 minutes. Tissues slides were than boiled in a steamer in citrate buffer (10mM citricacid monohydrat) ph6 for 30 minutes. After 20 minutes of cooling down, tissue on the glass slides were encircled with a fat-pen and first antibody (Anti CD98 (C-20): sc- 7095 (Santa Cruc Biotechnology, 1:1500)or Anti PCNA

- Proliferation Marker, 1: 2000 (Abcam, Cambridge, UK)) in a desired concentration was incubated on the tissue, for 30 minutes. Specimens were than washes with TBS before the second antibody (Anti Goat Immunglobulins/Biotinylated (Dako REAL, Glostrup, Denmark) or directly rabbit/mouse envisioner (Dako ) was added for 25 minutes. Again, after washing with TBS the DAB (Dako) according to manufactures instructions was incubated on the slides. DAB was then again removed by washing with TBS. After a short staining with Mayers Hemalaun-solution (Merck), slides were washed with water and incubated in HCl (0,44%)/ alcohol (70%) and washed with warm water. Slides were than covered with gel/mount for fixation and covered with a coverslip.

In vivo transplant assay

HighCD98hc/Caki2, lowCD98hc/ Caki2, silC98hc/ Caki2, poinsilCD98hc/Caki2 and trunsilCD98hc/Caki2 cells were xenotransplanted into nude mice by subcutaneous injection (s.c.) into the right flank, five animals per group. After 8 days mice were sacrificed and tumors were extracted and embedded in mounting medium. 4 micrometer sections were stained immunohistochemically for 4'-6-diamidino-2-phenylindole (DAPI), CD98hc ((C-20):

Cambridge, UK).
